# Rare pitfall in the magnetic resonance imaging of status epilepticus

**DOI:** 10.1016/j.ensci.2022.100405

**Published:** 2022-05-21

**Authors:** Mustafa Al-Chalabi, Silvi Bajrami, Nurose Karim, Ajaz Sheikh

**Affiliations:** a Department of Neurology, University of Toledo, Toledo, OH, USA; b College of Medicine and Life Sciences, University of Toledo, OH, USA

**Keywords:** FLAIR, Subarachnoid space, MRI pitfall, Status Epilepticus, HARM

## Abstract

Brain MRI in Status Epilepticus (SE) is often helpful in diagnosis, lateralization and localization of the seizure focus. MRI changes in SE include predominantly ipsilateral diffusion weighted imaging (DWI) changes in the hippocampus and pulvinar or similar changes involving basal ganglia, thalamus, cerebellum, brain stem and external capsule (Chatzikonstantinou et al., 2011 [1]). These changes are thought to be due to transient vasogenic and cytotoxic edema due to either transient damage or breakdown of blood brain barrier, proportional to the frequency and duration of the epileptic activity (Amato et al., 2001 [2]). Such changes may also be reflected on T2- weighted and T2-Fluid-Attenuated Inversion Recovery (FLAIR) sequences of MRI.

Herein, we present a case of a transient FLAIR cerebrospinal fluid (CSF) hyperintensity on the second MRI brain in a patient with focal status epilepticus. This imaging finding led to diagnostic confusion and was initially thought to represent subarachnoid hemorrhage. However, lumbar puncture, brain computed tomography (CT), and a follow-up brain MRI ruled out that possibility and other CSF pathologies. We concluded that the transient FLAIR changes in the second brain MRI were related to a rare imaging pitfall caused by Gadolinium enhancement of CSF on the FLAIR sequence, popularly referred to as hyperintense acute reperfusion marker (HARM).

## Highlights

• Postcontrast enhancement of the CSF on the FLAIR sequence can occur in seizures in a lateralizing fashion
• Transient FLAIR signal in subarachnoid spaces in seizure may lead to a diagnostic confusion with subarachnoid hemorrhage
• Renal insufficiency can precipitate this phenomenon in seizure patients
• Early recognition of this MRI pitfall is paramount to avoid unnecessary testing

## 1 Case presentation

**Fig. 1:**
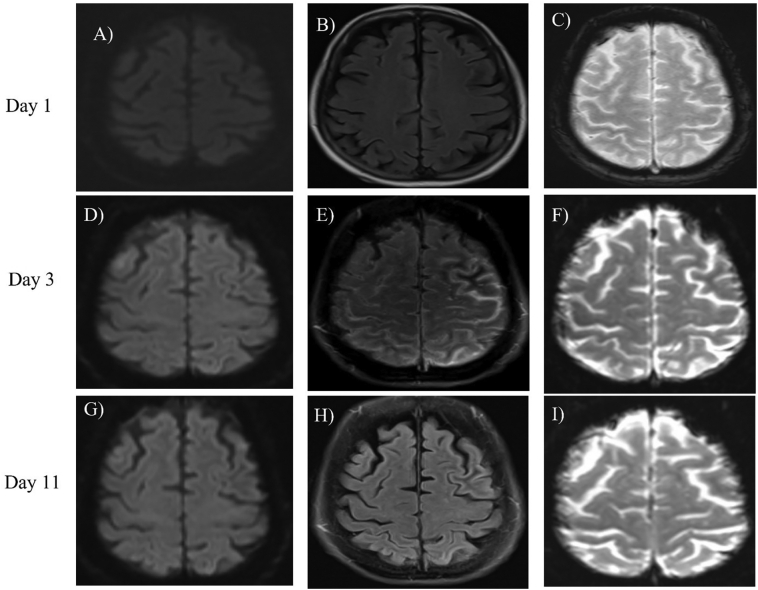
A–C: Axial images from brain MRI with three different sequences (DWI, FLAIR and GRE, respectively) at the time of presentation showing no abnormalities. D-F: Axial MRI images (in above sequence): Subarachnoid hyperintensity on FLAIR sequence (E) over the left parietal and frontal convexity with no corresponding changes on DWI (D) or GRE (F). G-I: Axial MRI images (in above sequence), showing complete resolution of the subarachnoid FLAIR hyperintensity (H) along the left parietal and frontal convexity with no abnormalities on DWI (G) or GRE (I).

**Fig. 2:**
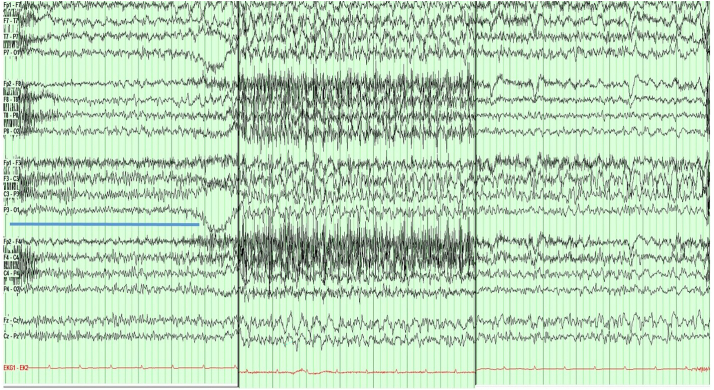
This composite picture shows the onset (left), organization (middle), and termination (right) of electrographic seizure. The seizure started in the left central region as 12–15 Hz rhythmic activity (underlined by blue bar). This was followed by organization, with spread into left temporal chain as 3 Hz semirhythmic activity, and appearance of 5–7 Hz sharp waves admixed with faster activity in left central region (middle panel). This is associated with dense EMG artifact on right side. Finally, the seizure terminated as 2–3 Hz polymorphic activity admixed with fast activity in the central region, over left hemisphere. Time base = 30 mm/s, Display sensitivity = 7 μV/div, Low frequency filter = 1 Hz and High frequency filter = 50 Hz. (For interpretation of the references to colour in this figure legend, the reader is referred to the web version of this article.)

A 71-year-old right-handed woman with past medical history of essential hypertension, hypothyroidism and stage-3 chronic kidney disease (baseline creatinine 1.2–1.5 mg/dl), who presented with repetitive episodes of altered mental status in the setting of severe hyperglycemia (serum glucose 436 mg/dl, HbA1C = 14.7%), related to new diagnosis of diabetes mellitus. These episodes lasted 90–120 s each, and were featured by incoherent speech and version of eyes and head to the right, followed by loss of consciousness and confusion for 3–5 min. Upon admission, the patient underwent a routine EEG recording that captured a prolonged electrographic seizure lasting about 5.5 min, and associated with right hand tonic posturing along with left-hand and oral automatisms. Initial brain MRI without and with 20 ml of Gadolinium (Gad) contrast on admission did not show any acute pathology (Fig. 1 A–C). She was started on Levetiracetam 1000 mg twice daily (maximum dose for her renal function) after an IV load, and was started on video EEG monitoring. The EEG detected multiple additional electrographic seizures arising from the left fronto-central region, each lasting 2.5–5 min, consistent with focal status epilepticus (Fig. 2). These were characterized by staring-off, version of the head and eyes to the right, and tonic posturing of right hand. Given the repetitive seizures, she required sequential addition of Fosphenytoin 150 mg three times a day, and Valproic acid (which was later discontinued due to hyperammonemia), followed eventually by sedation and intubation for the next 8 days. Midazolam and propofol infusions eventually controlled the seizures.

**Fig. 3:**
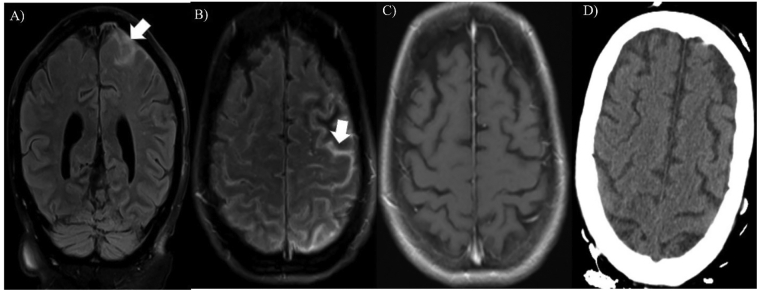
A–B: Coronal and axial FLAIR images, respectively, showing subarachnoid hyperintensity over the left parietal and frontal convexity. C: Axial T1 post-contrast image shows absence of any pathological enhancement. D: Axial brain CT image shows no evidence of subarachnoid blood.

Repeat brain MRI with 20 ml Gad contrast was performed two days after the first MRI, and showed localized subarachnoid hyperintensity on FLAIR sequence over the left parietal and frontal convexity, with no corresponding changes on DWI, gradient echo (GRE) (Fig. 1 D–F) or post contrast T1-weighted imaging (Fig. 3C). This pattern raised suspicion for subarachnoid blood. The patient was not on any anticoagulants and her coagulation profile was all within normal limits (protime 11 s, INR 1.0, and platelets 206 × 10^9^/l). This finding was perplexing, as the CT imaging of head at this time did not show any evidence of bleeding (Fig. 3D). The patient subsequently underwent lumbar puncture to fully rule out subarachnoid hemorrhage, which was largely unremarkable, with white blood cell count of 4/ul, zero RBCs, no xanthochromia, and mildly elevated protein at 61 mg/dl. The meningitis panel, cultures, and autoimmune epilepsy workup of CSF and blood were all negative. She underwent a third follow up MRI brain without and with Gad contrast, seven days after the second MRI, which showed a complete resolution of the previously mentioned FLAIR findings. She was discharged in stable condition on day 19, with diagnosis of status epilepticus secondary to severe hyperglycemia. She has followed-up in the outpatient clinic afterwards, with no further seizures, and a normal neurological examination.

## 2 Discussion

In status epilepticus, MRI changes include predominantly ipsilateral diffusion weighted imaging (DWI) changes in the hippocampus and pulvinar or similar changes involving basal ganglia, thalamus, cerebellum, brain stem and external capsule [1]. The transient vasogenic and cytotoxic edema due to either transient damage or breakdown of blood brain barrier, proportional to the frequency and duration of the epileptic activity, is thought to be the meachansim of these MRI changes [2]. FLAIR is an advanced MRI sequence that is designed to null the normal CSF signal. It has high sensitivity but low specificity for disease processes affecting the subarachnoid spaces, since any alteration in the CSF can result in increased signal on FLAIR imaging [3,4]. Hyperintensity in the subarachnoid space on FLAIR imaging was originally reported in 2004 with reperfusion injury and hemorrhagic transformation in the setting of ischemic stroke, hence termed in the current literature as “Hyperintense Acute Reperfusion Marker” (HARM) [5]. However, over the years multiple studies have described this as a nonspecific finding that is observed in a multitude of conditions, including subarachnoid hemorrhage, meningitis, stroke, transient ischemic attack [6], meningeal carcinomatosis, leptomeningeal metastasis, subdural hematoma, adjacent neoplasms, dural venous thrombosis and status epilepticus [3,7]. Additionally, this finding has also been linked to the use of supplemental oxygen in patients when undergoing MRI, and rarely following the administration of iodinated contrast material [3]. A 2021 retrospective observational study of 61 patients with MRI findings of HARM by Althaus et al., reported this finding in 35 patients with cerebrovascular disease, 12 patients with inflammatory CNS disease, and 14 patients with epilepsy. Their study, however, does not mention the indication for imaging in the epilepsy patients, and whether they had experienced any breakthrough seizures or status epilepticus prior to imaging. Further, interestingly, majority (10/14) of the epilepsy patients in their study had not received Gad based contrast previously [5].

Although current literature references the existence of a few case reports of this finding in epilepsy patients, upon deeper investigation, we were only able to find two reported cases by Villabolus-Chavez et al. [8], and Kim et.al [9]. On personal review of the articles, the imaging findings in the publication by Villabolus-Chavez et al. (which is the only other case of HARM changes post status epilepticus described in literature) were not strictly consistent with HARM, since changes were seen in other MRI sequences besides FLAIR. The case described by Kim et.al also did not have classical imaging findings of HARM only, as they described cortical hyperintensity and subcortical hypointensity on T-2 imaging, in addition to CSF FLAIR changes. In our case, this imaging finding was limited to the FLAIR sequence, was transient (not seen in the first and the third MRI) and lateralized to the left hemisphere, consistent with HARM. In strict technical terms, this makes ours the first reported case of classical HARM, in the absence of other typical imaging findings of SE, in a patient with electrographically confirmed focal status epilepticus.

With respect to the pathophysiology of HARM, multiple mechanisms of injury have been described [8]. Villabolos-Chavez et al., postulate in their case report that these findings could be explained by vasogenic and cytotoxic edema along with possible leptomeningeal and parenchymal uptake of the Gad contrast injection, following SE induced BBB breakdown over the side of seizure [8]. This process may be further complicated by the prolonged clearance of Gad chelate due to reduced glomerular filtration rate. One hypothesis is that in the setting of renal insufficiency with concurrent prolonged elevation of Gad concentration, Gad may move across an osmotic gradient [3]. Bozzao et al. [4] concluded that the administration of IV contrast 2–24 h prior to the acquisition of FLAIR images is directly correlated with aberrant CSF signals in the FLAIR sequences when there is disease altering blood brain barrier near the subarachnoid spaces such as stroke or neoplasm [4,10]. The fact that these findings were resolved in eight days further supports its association with prolonged Gad retention. However, interestingly, the study by Althaus et al., demonstrated no clear association between HARM and pretreatment with Gad and/or recanalization therapies in ischemic stroke patients, challenging the notion that HARM is somehow related to Gad leakage and retention in the CSF spaces or to reperfusion injury [5]. The authors of that study proposed renaming this phenomenon as **FLA**IR **S**ubarachnoid **H**yperintensity, or FLASH, to account for the broad multitude of underlying diseases and mechanisms of damage linked to this phenomenon [5].

Notwithstanding the lack of perfect understanding of the exact mechanism of this interesting phenomenon, we believe that the neurologists and neuroradiologists should be aware of this potential imaging pitfall in epilepsy (including status epilepticus) patients, as it could lead to diagnostic confusion and further unnecessary tests.

## Declaration of Competing Interest

On behalf of all authors, the corresponding author states that there is no conflict of interest. On behalaf of all authors, the corresponding authors acknowledge the department of neurology at The 10.13039/100012569University of Toledo for funding this research.
